# Strategies for Antithrombotic Management During Non-cardiac Arterial Procedures: Results of the International ACTION Survey

**DOI:** 10.1016/j.ejvsvf.2025.01.005

**Published:** 2025-04-02

**Authors:** Max Hoebink, Vincent Jongkind, European Vascular Research Collaborative, Charlotte Arendt, Gert J. de Borst, Albert Busch, Caroline Caradu, Alexander Croo, Vaiva Dabravolskaité, Mariam Darwish, Mario D'Oria, Harm P. Ebben, Florian Enzmann, Qasam M. Ghulam, Alexander Gombert, Alexandra Gratl, Bergros K. Johannesdottir, Angelos Karelis, Aoife Kiernan, Leszek Kukulski, Fabien Lareyre, Ryan Gouveia e Melo, Cecilie Markvard Møller, Panagiotis Doukas, Nikolaos Patelis, Konstantinos Spanos, Paolo Spath, Martin Teraa, Bich L. Tran, Christian Zielasek, Petar Zlatanovic, Kak Khee Yeung, ACTION-1 Research Collaborative, Liliane C. Roosendaal, Jan D. Blankensteijn, Hessel C.J.L. Buscher, Daniël Eefting, Bram Fioole, Jan M.M. Heyligers, Rutger J. Hissink, Rigo Hoencamp, Mark J.W. Koelemay, Rogier H.J. Kropman, Lijckle van der Laan, Susan Lemson, Maurice E.N. Pierie, Boudewijn L. Reichmann, Michel M.P.J. Reijnen, Jan van Schaik, Peter M. Schlejen, Joep A.W. Teijink, Edith M. Willigendael, Clark J. Zeebregts, Arno M. Wiersema

**Affiliations:** a Amsterdam UMC location University of Amsterdam, Vascular Surgery, Meibergdreef 9, Amsterdam, the Netherlands; b Dijklander Ziekenhuis, Vascular Surgery, Maelsonstraat 3, Hoorn, the Netherlands; c Amsterdam Cardiovascular Sciences, Atherosclerosis and Aortic Syndromes, Amsterdam, the Netherlands

**Keywords:** Activated clotting time, Antithrombotic strategies, Heparin, Non-cardiac arterial procedures, Protamine, Vascular surgery

## Abstract

**Objective:**

Peri-procedural antithrombotics are used extensively to prevent thromboembolic complications during non-cardiac arterial procedures (NCAP) worldwide. However, there is a lack of evidence to support recommendations on antithrombotic strategies, possibly leading to substantial variation in local practices. A comprehensive overview of antithrombotic strategies is needed to identify the most widely accepted protocols employed during NCAP, highlight variations in local practices, and identify new research targets to establish evidence based peri-procedural anticoagulation management.

**Methods:**

An international, web based survey study was conducted from March to October 2023, targeting vascular clinical specialists who applied antithrombotic strategies during NCAP in daily practice.

**Results:**

The survey was completed by 436 vascular clinical specialists from 45 countries (Europeans: 93%, vascular surgeons or vascular surgery residents: 98%). Systemic unfractionated heparin was used by nearly all vascular specialists during all procedures (varying between 98–99%, depending on the procedure type), but could vary depending on specific NCAP. A fixed starting dose (39–52%, most often 5 000 IU [80–89%]) or an actual bodyweight dependent dose (42–52%, most commonly 100 IU/kg [40–67%] or 50 IU/kg [17–40%]) was mainly used. Except during fenestrated or branched endovascular aneurysm repair procedures (51%), activated clotting time (ACT) was employed by a minority (26–31%). A large variety in measurement protocols was observed, yet a target ACT of 200 seconds was most often used for all NCAP types (44–54%). Most vascular specialists considered a heparin follow up dose (61–81%) and heparin reversal using protamine (54–63%), both for a variety of indications. Of the participants, 68% expressed discontent with their current antithrombotic protocol(s).

**Conclusion:**

This comprehensive, international survey study revealed large variation among vascular clinical specialists’ heparinisation strategies during NCAP. Together with the considerable discontent expressed regarding protocols, this emphasises the urgent need for comparative, randomised studies on antithrombotic management during NCAP.

## Highlights

• An international survey on antithrombotic strategies during non-cardiac arterial procedures was undertaken.
• Four hundred and thirty-six vascular clinical specialists from 45 countries completed the survey.
• There was a large variation in heparin dosing, heparin monitoring, and heparin reversal strategies.
• Considerable discontent was expressed regarding the existing protocols.
• High quality studies on peri-procedural antithrombotic management are needed.

## INTRODUCTION

Antithrombotic strategies are commonly applied during non-cardiac arterial procedures (NCAP) to reduce thromboembolic complications such as peripheral emboli, graft thrombosis, intestinal ischaemia, and myocardial infarction, and achieve safe and effective treatment.^1^, ^2^, ^3^ Unfractionated heparin is the most used antithrombotic agent, but its efficacy is unpredictable in the individual patient, which could complicate maintaining adequate anticoagulation levels.^4^^,^^5^ To monitor the heparin effect and regulate follow up heparin dosages, activated clotting time (ACT) is being used increasingly during NCAP.^1^^,^^6^ The risk of bleeding complications may be lowered by reversing heparin after removal of arterial clamps or endovascular sheaths to restore coagulation at the end of surgery using protamine.^7^

While heparin has been part of worldwide standard practice during NCAP for over 70 years, detailed evidence of its effectiveness is lacking.^6^^,^^8^^,^^9^ Consequently, clinical practice guidelines do not provide recommendations on heparinisation strategies or lack robust support by evidence.^10^, ^11^, ^12^, ^13^, ^14^, ^15^ This paucity of guidance by literature and guidelines might have led to substantial differences in locally implemented antithrombotic strategies. A Dutch nationwide survey showed considerable variability in peri-procedural anticoagulation strategies.^1^ Such variability might extend beyond national borders, yet recent overviews from other countries or international cohorts are missing.^16^, ^17^, ^18^ An international study on the currently used antithrombotic strategies during NCAP may guide vascular specialists and highlight international differences. Potential knowledge gaps and variations in local practices might be identified, supporting the need and trajectory of future trials to establish evidence based peri-procedural anticoagulation management. Therefore, a comprehensive international survey study was conducted to provide broad insight into the currently used antithrombotic strategies during NCAP.

## METHODS

### Study design

An international survey study was conducted on peri-procedural antithrombotic strategies during NCAP in collaboration with the European Vascular Research Collaborative (EVRC, eurovascresearch.com) and the ACTION-1 Research Collaborative (ACTION-1 trial: clinicaltrials.gov: NCT04061798). This was an open access, voluntary, population based survey targeting vascular clinical specialists coordinating antithrombotic strategies during NCAP; they could be consultant (cardio)vascular surgeons or anaesthetists. In this report, participants involved in the study are referred to as vascular specialists.

### Survey construction

The survey was constructed and deployed using the web based survey platform SurveyMonkey (SurveyMonkey Inc., San Mateo, CA, USA, surveymonkey.com). Questions were written in English. Questions were stratified for the following NCAP types: carotid endarterectomy (CEA), (thoracic) endovascular aneurysm repair (EVAR and TEVAR), fenestrated or branched endovascular aneurysm repair (F-EVAR or B-EVAR), open abdominal aortic repair (OAR), and peripheral arterial disease surgical procedures (PAD surgery). Peripheral arterial disease surgery could involve femoral endarterectomy, peripheral bypass surgery, or hybrid procedures. Endovascular peripheral vascular procedures were not included. The survey consisted of 74 questions on peri-procedural anticoagulation and focused on unfractionated heparin administration, monitoring by ACT and APTT, and reversal using protamine. General information regarding hospital type, country of employment, and satisfaction with the currently used antithrombotic strategies were also gathered. Features were incorporated into the survey design to allow participants to bypass questions if they were irrelevant, based on previously provided answers, or if antithrombotic protocols were identical to previously completed protocols for other NCAP types. To minimise missing responses, the survey design incorporated a requirement for participants to answer each question before continuing the survey. To ensure that questions were clear and internationally applicable, a pilot version of the survey was sent to an international group of 12 collaborators and feedback was incorporated into the definitive survey design. The complete survey is displayed in Supplementary material S1. Mean completion time of the survey was 7 minutes.

### Survey deployment

The survey was conducted from March to October 2023 and was accessible via a web based link or QR code. Survey invitations were repeatedly disseminated by collaboration group members via social media, email, and during scientific conferences. The survey was accessible via the EVRC and ACTION-1 websites. Invitations were also disseminated by national vascular surgical societies. The General Data Protection Regulation (GDPR) was employed when handling data of participating vascular specialists.^19^ In addition, IP restriction was implemented, enabling participants to only complete one survey per IP address. Received responses were individually validated based on contact information provided by participants. Data were encrypted and stored in the Amsterdam UMC database. A participant key file was secured with a password only known to the coordinating investigator (MH) and stored in a separate location within the database.

### Statistical analysis

Statistical Package for the Social Sciences (SPSS) version 28 (IBM Corp., Armonk, NY, USA) was used for data analyses. Data were screened manually for double entries from the same participant based on contact details, prematurely terminated surveys, missing answers to optional questions, and clearly invalid data entries. Double entry data were removed manually before analyses. Data from prematurely ended surveys were included into data analysis when deemed valid. Questions with missing answers were excluded separately. Only reporting general information without reporting antithrombotic strategies, extreme initial heparin dosing values (<2 000 IU, <30 IU/kg, >15 000 IU, >200 IU/kg), or performing follow up ACT measurements ≤5 minutes after reaching target ACT were considered invalid data entries. Continuous variables were expressed as medians and interquartile ranges (IQR), and categorical variables as counts and percentages. Descriptive analyses were performed. Based on the specific measurement equipment used, Kruskal–Wallis tests were used to analyse potential differences in used target ACTs.^20^ *P* values <0.050 were considered statistically significant.

## RESULTS

### General information

Four hundred and sixty-three participants initiated the survey. After removal of duplicate and invalid data entries, 436 vascular medical specialists from 45 different countries were included. Of these vascular specialists, 406 (93%) were practicing in European hospitals. Inclusions per country are listed in Supplementary Table S1. Vascular specialists were employed in various hospital types: 264 (61%) university or tertiary care hospital, 108 (25%) peripheral or secondary care hospital, 37 (8%) combined employment of tertiary and secondary care hospital, 21 (5%) private hospital, and six (1%) other institutes. Most participating vascular specialists (425) were vascular surgeons or vascular surgery residents (98%), followed by five anaesthetists or anaesthetic residents (1%), five angiologists (1%), and one interventional cardiologist (<1%). Seven (2%) participants were residents (six vascular surgery residents and one anaesthetics resident). Since data received from vascular surgery residents and anaesthetic residents were very limited and the protocols used were most probably initiated by vascular surgeons and anaesthetists, respectively, subgroups for residents were not established. Median experience of the included participants in their clinical profession was 10 years (IQR 5, 15).

### Antithrombotic strategies

The number of responses reported per procedure type were: 419 CEA, 348 EVAR or TEVAR, 204 F-EVAR or B-EVAR, 370 OAR, and 378 PAD surgery. Systemic heparin was used by most vascular specialists: 412 (98%) CEA, 343 (99%) EVAR or TEVAR, 203 (99%) F-EVAR or B-EVAR, 365 (99%) OAR, and 372 (98%) PAD surgery. One vascular specialist (<1%) exclusively administered local arterial heparin in the extremities during OAR. Low molecular weight heparin instead of heparin was used as the antithrombotic agent by a fraction of vascular specialists: seven (2%) CEA, five (1%) EVAR or TEVAR, one (<1%) F-EVAR or B-EVAR, five (1%) OAR, and five (1%) PAD surgery. Two vascular specialists (<1%) reported to administer 250 mg of intravenous aspirin during PAD surgery instead of heparin. One vascular specialist (<1%) reported not using antithrombotics during OAR, and one vascular specialist (<1%) did not use antithrombotics during PAD surgery. Protocols without using systemic heparin were all practiced by European vascular specialists.

### Heparin dosing

Details on heparin dosing are displayed in Table 1, Table 2. Ten heparin starting doses were considered to be extreme values and were excluded. An intravenous fixed starting dose was used during most procedures (39–52%, depending on NCAP type). Primarily this was a bolus of 5 000 IU (80–89%). Dosing based on actual bodyweight was also frequently practiced, most often during F-EVAR or B-EVAR (52%). A minority (4–6%) based heparin dose on ideal bodyweight. Three vascular specialists (<1%) mentioned increasing their starting dose (100 IU/kg instead of 50 IU/kg) during CEA if shunting was needed. Most vascular specialists considered administering follow up doses of heparin (61–81%). Indications for follow up dosing varied between NCAP types (Table 3).

**Table 1:** Fixed heparin starting dose strategies per type of non-cardiac arterial procedure.

Procedures	Starting doses∗	<2 500 IU	2 500–4 999 IU	5 000 IU	5 001–7 500 IU	7 501–10 000 IU	Variable dose
CEA	215 (52)	1 (<1)	29 (13)	171 (80)	3 (1)	10 (5)	1 (<1)
EVAR/TEVAR	167 (49)	–	12 (7)	149 (89)	2 (1)	3 (2)	1 (<1)
F-EVAR or B-EVAR	79 (39)	–	6 (8)	68 (86)	3 (4)	2 (2)	–
OAR	180 (49)	1 (<1)	23 (13)	151 (84)	1 (<1)	3 (2)	1 (<1)
PAD surgery	184 (50)	1 (<1)	22 (12)	157 (85)	–	3 (2)	1 (<1)

Data are shown as *n* (%).

CEA = carotid endarterectomy; EVAR/TEVAR = endovascular aneurysm repair and or thoracic endovascular aneurysm repair; F-EVAR or B-EVAR = fenestrated endovascular aneurysm repair and or branched endovascular aneurysm repair; OAR = open abdominal aortic repair; PAD = peripheral arterial disease.

∗ Starting dose strategies are depicted as percentages of total heparin starting dose strategies included.

**Table 2:** Bodyweight dependent heparin starting dose strategies per type of non-cardiac arterial procedure.

Procedures	Based on actual bodyweight							Based on ideal bodyweight		
	Starting dose∗	<50 IU/kg	50 IU/kg	51–99 IU/kg	100 IU/kg	>100 IU/kg	Variable dose	Starting doses∗	50–99 IU/kg	100 IU/kg
CEA	171 (42)	3 (2)	58 (34)	24 (14)	75 (44)	9 (5)	2 (1)	19 (5)	6 (32)	13 (68)
EVAR/TEVAR	149 (43)	1 (<1)	54 (36)	25 (17)	67 (45)	2 (1)	–	17 (5)	8 (47)	9 (53)
F-EVAR or B-EVAR	105 (52)	1 (<1)	18 (17)	14 (13)	70 (67)	2 (2)	–	12 (6)	6 (50)	6 (50)
OAR	159 (44)	3 (2)	63 (40)	24 (15)	64 (40)	3 (2)	2 (1)	15 (4)	5 (33)	10 (67)
PAD surgery	164 (44)	3 (2)	66 (40)	22 (13)	70 (43)	2 (1)	1 (<1)	19 (5)	7 (37)	12 (63)

Data are shown as *n* (%).

CEA = carotid endarterectomy; EVAR/TEVAR = endovascular aneurysm repair and or thoracic endovascular aneurysm repair; F-EVAR or B-EVAR = fenestrated endovascular aneurysm repair and or branched endovascular aneurysm repair; OAR = open abdominal aortic repair; PAD = peripheral arterial disease.

∗ Starting dose strategies are depicted as percentages of total heparin starting dose strategies included.

**Table 3:** Primary heparin follow up dose indications per type of non-cardiac arterial procedure.

Procedures	Responses	No follow up dose	Primarily based on ACT	Primarily based on APTT	Primarily based on subjective coagulation status/length of surgery	According to a standardised protocol (not based on ACT)	Yes, by continuous heparin infusion	Other∗ (%)
CEA	406	157 (39)	100 (24)	2 (<1)	98 (24)	41 (10)	1 (<1)	7 (2)
EVAR/TEVAR	342	109 (32)	96 (28)	2 (<1)	96 (28)	33 (10)	1 (<1)	5 (1)
F-EVAR or B-EVAR	202	38 (19)	95 (47)	1 (<1)	38 (19)	25 (12)	4 (2)	1 (<1)
OAR	365	113 (31)	89 (24)	1 (<1)	120 (33)	37 (10)	1 (<1)	4 (1)
PAD surgery	372	114 (31)	83 (22)	2 (<1)	125 (34)	39 (10)	1 (<1)	8 (2)

Data are shown as *n* and *n* (%).

ACT = activated clotting time; APTT = activated partial thromboplastin time; CEA = carotid endarterectomy; EVAR/TEVAR = endovascular aneurysm repair and or thoracic endovascular aneurysm repair; F-EVAR or B-EVAR = fenestrated endovascular aneurysm repair and or branched endovascular aneurysm repair; OAR = open abdominal aortic repair; PAD = peripheral arterial disease.

∗ Other: other indications, variable primary indications, or not reported.

### Heparin monitoring

Except during F-EVAR or B-EVAR procedures, a minority of vascular specialists used ACT to monitor the heparin effect: 113 (27%) CEA, 107 (31%) EVAR or TEVAR, 104 (51%) F-EVAR or B-EVAR, 98 (27%) OAR, and 95 (26%) PAD surgery. Data on ACT target values are expressed in detail in Table 4. There was substantial variation in the target ACTs used, yet a target ACT of 200 seconds was most often used for all NCAP types (44–54%). Timing of ACT measurements is shown in Supplementary Table S2. Most vascular specialists performed a measurement during the first five minutes after heparin administration for most NCAP types (21–43%), and almost always within 30 minutes after heparin administration (84–90%). Four follow up ACT measurements were performed ≤5 minutes after reaching target ACT and were considered invalid. When the target ACT was reached, a follow up measurement was usually performed after 30 minutes (40–46%) or 60 minutes (35–43%). A minority of respondents using ACT employed a target ACT value for the end of the procedure: 23 (20%) CEA, 26 (24%) EVAR or TEVAR, 26 (25%) F-EVAR or B-EVAR, 27 (37%) OAR, and 23 (24%) PAD surgery (Table 4).

**Table 4:** Activated clotting time target values per type of non-cardiac arterial procedure.

Procedure	Target ACT during surgery – seconds							Target ACT at closure – seconds				
	ACT users∗	150–199	200	201–250	>250	2 times baseline value	Both fixed target value and 2 times baseline value	ACT users∗	≤150	151–200	201–250	>250
CEA	79 (70)	8 (10)	43 (54)	19 (24)	3 (4)	2 (3)	4 (5)	23 (20)	9 (39)	9 (39)	5 (22)	–
EVAR/TEVAR	73 (68)	10 (14)	36 (49)	22 (30)	2 (3)	3 (4)	–	26 (24)	9 (35)	10 (38)	7 (27)	–
F-EVAR or B-EVAR	77 (74)	5 (6)	35 (46)	26 (34)	5 (6)	4 (5)	2 (3)	26 (25)	5 (19)	12 (46)	7 (27)	2 (8)
OAR	74 (76)	11 (15)	40 (54)	16 (21)	2 (3)	2 (3)	3 (4)	27 (28)	9 (33)	12 (44)	5 (19)	1 (4)
PAD surgery	23 (24)	3 (13)	10 (44)	4 (17)	2 (9)	4 (17)	–	23 (24)	8 (35)	10 (43)	5 (22)	–

Data are shown as *n* (%).

ACT = activated clotting time; CEA = carotid endarterectomy; EVAR/TEVAR = endovascular aneurysm repair and or thoracic endovascular aneurysm repair; F-EVAR or B-EVAR = fenestrated endovascular aneurysm repair and or branched endovascular aneurysm repair; OAR = open abdominal aortic repair; PAD = peripheral arterial disease.

∗ Target ACT values are depicted as percentage of total of ACT users per procedure.

The measurement device used was reported by 138 (78%) vascular specialists who used ACT for one or more NCAP type(s). Other participants did not know (18, 10%) or did not list the used device (22, 12%). Devices that were used were: 60 (44%) ACT Plus, Medtronic, 26 (19%) Hemostasis Management System Plus, Medtronic, 23 (17%) I-STAT, Abbot, 22 (16%) Hemochron Signature Elite, six (4%) Hemochron Response, and one (<1%) Actalyke mini ii, Helena Laboratories. Kruskal–Wallis tests did not show statistically significant differences in target ACTs based on the measurement device used for all NCAP types: CEA *p* = 0.42; EVAR or TEVAR *p* = 0.45; F-EVAR or B-EVAR *p* = 0.32; OAR *p* = 0.66; and PAD surgery *p* = 0.88.

A fraction of vascular specialists primarily used APTT to monitor heparin: two CEA, two EVAR or TEVAR, one F-EVAR or B-EVAR, one OAR, and two PAD surgery (all <1%).

### Protamine administration

Most vascular specialists considered protamine use during NCAP: 242 (63%) CEA, 181 (54%) EVAR or TEVAR, 111 (56%) F-EVAR or B-EVAR, 216 (60%) OAR, and 200 (54%) PAD surgery. Details on primary indications for protamine administration are provided in Table 5.

**Table 5:** Primary indication to administer protamine per type of non-cardiac arterial procedure.

Procedure	Responses	No protamine	Based on subjective coagulation status/length of surgery	Based on amount of heparin administered	Based on ACT at closure	Other∗
CEA	384	142 (37)	128 (33)	67 (18)	34 (9)	13 (3)
EVAR/TEVAR	337	156 (46)	99 (29)	40 (12)	32 (10)	10 (3)
F-EVAR or B-EVAR	197	86 (43)	51 (26)	29 (15)	25 (13)	6 (3)
OAR	362	146 (40)	119 (33)	52 (15)	34 (9)	11 (3)
PAD surgery	370	170 (46)	110 (30)	52 (14)	29 (8)	9 (2)

Data are shown as *n* and *n* (%).

ACT = activated clotting time; CEA = carotid endarterectomy; EVAR/TEVAR = endovascular aneurysm repair and or thoracic endovascular aneurysm repair; F-EVAR or B-EVAR = fenestrated endovascular aneurysm repair and or branched endovascular aneurysm repair; OAR = open abdominal aortic repair; PAD = peripheral arterial disease.

∗ Always, other indications, variable primary indications, or not reported.

### Protocol satisfaction

Of the 382 vascular specialists (88%) who commented on potential improvement in their antithrombotic protocol(s), 121 (32%) were satisfied with their current antithrombotic strategies. The remaining 54 (12%) vascular specialists did not list an answer regarding protocol satisfaction. A preference for a more patient tailored protocol(s) was expressed by 168 (44%). Additionally, 129 (34%) of the vascular specialists expressed a desire to monitor the effect of heparin intra-operatively using ACT or to broaden their indications for ACT use. One vascular specialist (<1%) mentioned that heparin might not be needed at all during NCAP types.

## DISCUSSION

This survey study provides a comprehensive, international overview regarding antithrombotic strategies used during CEA, EVAR or TEVAR, F-EVAR or B-EVAR, OAR, and PAD surgery. The participating vascular specialists stated that they use systemic unfractionated heparin during nearly every procedure. Overall, substantial variation was found in applied strategies regarding heparin dosing, ACT guided monitoring, and protamine administration during NCAP.

The considerable variation that was seen seems understandable, since previous systematic reviews on prophylactic antithrombotics and ACT use during NCAP showed that compelling evidence is still missing.^6^^,^^8^^,^^9^ Only one small randomised trial in NCAP has been performed supporting peri-operative heparin use during open abdominal aortic aneurysm repair.^2^ Consequently, clinical practice guidelines lack well founded and specific recommendations, and antithrombotic management during NCAP is open to local preference based on clinical experience.^12^, ^13^, ^14^, ^15^ The large variation in antithrombotic strategies could also mean that multiple protocols are sufficiently effective, and that homogenising antithrombotic strategies does not further minimise peri-procedural thromboembolic and bleeding complications. However, common guidelines generally improve patient outcomes, promote consistency in care, and, if publicly available, increase transparency of medical decision making.^21^^,^^22^ Additionally, most vascular specialists (68%) expressed discontent with their existing antithrombotic protocols. Hence, comparative studies should be performed to identify the most effective, safe, and cost-efficient antithrombotic strategy during NCAP.

Fixed *vs*. actual bodyweight dependent dosing could serve as a target for future comparative research, since there was a dichotomy between the use of these regimens in the current study (39–52% *vs*. 42–52%, respectively). Previous research has suggested administering an actual bodyweight dependent dose of 100 IU/kg heparin over a fixed dose of 5 000 IU to achieve adequate anticoagulation (i.e. ACT 200–250 seconds), but these were non-comparative, observational studies.^4^^,^^23^ Also, since the ACT measurement protocols used by vascular specialists varied substantially, its use during NCAP should be specified. A follow up study of the aforementioned observational studies compared ACT guided heparinisation using a starting dose of 100 IU/kg heparin and a target ACT of 200 seconds with a standardised dose of 5 000 IU heparin in a propensity score matched NCAP cohort.^24^ The incidence of thromboembolic complications (6.2% *vs*. 4.8%; *p* = 0.52), death (1.4% *vs*. 0 patients; *p* = 0.25), and bleeding complications (15% *vs*. 12%; *p* = 0.32) was similar between groups. These findings indicate that ACT guided heparinisation using this protocol is not superior during NCAP. The abovementioned ACT guided heparinisation protocol is currently being compared with a single heparin dose of 5 000 IU during open abdominal aortic aneurysm surgery in the first randomised trial on ACT guided heparinisation (ACTION-1 trial, clinicaltrials.gov: NCT04061798).^23^^,^^25^ Results from this trial are eagerly awaited. Additionally, future studies should aim to develop standardised protocols for protamine administration. In a meta-analysis, protamine use was found to reduce the risk of wound haematoma and wound haematoma requiring re-operation, without increasing the procedural stroke rate during CEA.^7^ Due to a large variety of included protamine strategies, an optimal strategy could not be deduced, and protamine use has not been proven beneficial for other NCAP. Lastly, future studies could include comparing systemic heparin with local heparin or other antithrombotic agents. In the present study, a fraction of participants did not administer systemic heparin, but used local heparin (<1%), IV aspirin (<1%), low molecular weight heparin (1–2%, depending on NCAP type), or no antithrombotics at all (<1%) during specific NCAP. However, since this was a voluntary open access survey, the incidence of vascular specialists that do not administer systemic heparin might have been underestimated.

The optimal heparin strategy may differ depending on the type of NCAP. Increased bleeding risk in relation to heparin in patients undergoing percutaneous peripheral interventions and undergoing CEA were described at variable heparin dosages (>60 IU/kg and >90 IU/kg, respectively).^26^^,^^27^ Additionally, when employing ACT guidance, the heparinisation strategy potentially differs based on the measurement equipment used. In the current study, vascular specialists reported a large variation in ACT measurement devices, but there was no association between the measurement devices used and the target ACTs defined. Differences of up to 63 seconds are described between measurement devices, suggesting adjusting ACT monitoring protocol(s) to the monitoring equipment used.^28^ Thus, the optimal heparin dosing regimen might not be reduced to a single protocol.

In the current study, weight based heparin administration, follow up dosing, and ACT guided monitoring were frequently used during F-EVAR or B-EVAR compared with other NCAP (51%). A relatively low risk of bleeding combined with the deleterious risks of graft thrombosis in small calibre branches used during these procedures could explain the increased attention to maintaining a high heparin anticoagulation level. Interestingly, a previous Delphi study on antithrombotic strategies during F-EVAR or B-EVAR settled on using ACT guided heparinisation, targeting an ACT of 250–300 seconds.^29^ Most vascular specialists in the present study targeted an ACT of 200–250 seconds (80%). As the Delphi design implicates, recommendations of the aforementioned study were based on expert opinion, and evidence to support these recommendations is still required. Yet, different views on optimal target ACTs apparently exist, emphasising antithrombotic management during F-EVAR or B-EVAR procedures as another possible research target.

This overview has the potential to serve as a reference on heparinisation strategies for vascular specialists performing NCAP based on international expert opinion, which is awaiting high quality evidence. Results of this survey could offer guidance to researchers initiating follow up research regarding antithrombotic management during NCAP.

### Limitations

Several limitations should be acknowledged. The survey was intentionally kept concise to minimise missing data and did not include antithrombotic strategies used during peripheral endovascular interventions, and dose specifications of heparin follow up dosages or protamine administrations. Data on specific heparin dosages based on ACT guided measurements were collected but were highly inconsistent and incomplete due to question formulation and thus were not included in this report. The open access design of this survey could have introduced non-response bias, directing towards specific vascular specialists and preferences. Also, the design could have led to unreliable answers, but credibility of participating vascular specialists was manually verified based on the contact details provided to mitigate this risk. Participating vascular specialist were almost exclusively European vascular surgeons, possibly reducing generalisability to other medical professions or continents. Additionally, using a single language enhanced the risk of question misinterpretation. A pilot version of the survey was distributed to an international group of collaborators for feedback before data collection to minimise ambiguously interpretable questions.

### Conclusions

This survey has provided a comprehensive, international overview of peri-procedural antithrombotic strategies during NCAP. The findings revealed substantial variation in antithrombotic management regarding heparin dosing, ACT guided monitoring, and protamine administration. Together with a considerable number of vascular specialists expressing discontent with their current antithrombotic protocols, this emphasises the urgent need for comparative, randomised studies on antithrombotic management in vascular surgery.

## Funding

This research did not receive any specific grant from funding agencies in the public, commercial, or not for profit sectors.

## Data statement

Data has not been made publicly available.

## Conflict of interest

None.
