# Supplementary material for: Strategies for Antithrombotic Management During Non-cardiac Arterial Procedures: Results of the International ACTION Survey
Source: EJVES Vasc Forum. 2025 Apr 2;64:8–15. doi: 10.1016/j.ejvsvf.2025.01.005 (PMC12269446; doi:10.1016/j.ejvsvf.2025.01.005)
Supplement: Multimedia component 1 [file mmc1.docx]

**Supplementary material**

**Supplementary material S1.** The complete survey outline.

**Page 1 Contact details**

1. What is your profession?
   1. Vascular surgeon
   2. Interventional radiologist / angiologist
   3. Interventional cardiologist
   4. Anaesthesiologist
   5. Other (please specify)
2. How many years of experience do you have in this profession? (fill in the number of years)
3. In which country do you work?
4. In which hospital do you work?
5. In what kind of hospital do you work?
   1. Academic / tertiary care hospital
   2. Peripheral / secondary care hospital
   3. Academic and peripheral hospital
   4. Other (please specify)
6. What is your E-mail address?

**Questions per type of procedure (CEA, EVAR/TEVAR, FEVAR/BEVAR, open abdominal aortic repair, peripheral arterial surgical procedures)**

**Page 2 Antithrombotic strategy (fill in per type of procedure)**

1. What kind of antithrombotic strategy do you use during …?
   1. I do not perform this procedure (go to next procedure)
   2. I use the same antithrombotic strategy as during … (go to next procedure)
   3. I use a different antithrombotic strategy

**Page 3 Heparin dose (fill in per type of procedure)**

1. What kind of IV anticoagulant do you administer during the procedure?
   1. A fixed starting dose of heparin (example: 5 000 IU)
      1. Explain how many international units (IU) (fill in a whole number) …
   2. A heparin dose based on **actual** bodyweight (example 100 IU/kg)
      1. Explain how many international units (IU)/kg (fill in a whole number) ...
   3. A heparin dose based on **ideal** bodyweight (example 100 IU/kg)
      1. Explain how many international units (IU)/kg (fill in a whole number) ...
   4. I use LMWH instead of unfractionated heparin (go to next procedure)
   5. I do not use heparin (go to next procedure)
   6. Other heparin starting dose (please specify, to next procedure)
2. Do you consider giving additional unfractionated heparin after the starting dose?
   1. No (go to protamine)
   2. Yes, a fixed dose according to a standardized protocol (not based on ACT) (go to protamine)
   3. Yes, a dose depending on bodyweight, according to a standardized protocol (not based on ACT) (go to protamine)
   4. Yes, by using a continuous heparin perfusor (with or without ACT) (go to continuous heparin perfusion)
      1. How much heparin do you administer by continuous perfusion after the starting dose? (explain how many international units (IU)/hour or IU/kg/hour) …
      2. Do you adjust continuous heparin perfusion based on ACT?
         1. Yes (go to ACT)
         2. No, based on APTT (go to protamine)
         3. No, bases on (please specify) (go to protamine)
   5. Yes, primarily based on ACT (go to ACT)
   6. Yes, primarily based on APTT (go to protamine)
   7. Yes, a fixed dose, primarily based on subjective coagulation status and/or length of surgery (not based on ACT) (go to protamine)
   8. Yes, a dose depending on bodyweight, primarily based on subjective coagulation status and/or length of surgery (not based on ACT) (go to protamine)
   9. Other (please specify) … (go to protamine)

**Page 4 Activated Clotting Time (fill in per type of procedure)**

1. What is your target ACT? (choose 1 option)
   1. … seconds (minimally acceptable value)
   2. … OR: how many times the reference value
2. How many minutes after heparin administration do you perform the first ACT measurement? (fill in a whole number)
   1. … seconds
3. How many minutes after reaching the target ACT do you measure the ACT again? (fill in a whole number)
   1. … seconds
4. How much additional heparin do you administer? (explain how many IU of heparin at which ACT)
   1. … heparin (IU of IU/kg) at ACT (sec)
   2. … heparin (IU of IU/kg) at ACT (sec)
   3. … heparin (IU of IU/kg) at ACT (sec)
5. What is the target ACT at the end of the procedure?
   1. I don’t have a target ACT at the end of the procedure
   2. Fill in the maximum acceptable value in seconds …

**Page 5 Protamine (fill in per type of procedure)**

1. What is the primary indication to give protamine?
   1. I do not administer protamine during this type of procedure
   2. Based on subjective intra-operative coagulation status and/or length of surgery
   3. Based on the amount of heparin administered
   4. Based on ACT at closure
   5. Other indication (explain) …

**Page 6 General questions**

1. Would you like to improve something on your current antithrombotic protocol(s) during NCAP?
   1. No
   2. I would like protocol(s) to be more tailored to the individual patient
   3. I would like to monitor the effect of heparin intraoperatively by ACT
   4. Other (explain) …
2. If you measure ACT, which device do you use to measure the ACT during NCAP?
   1. I do not monitor the effect of heparin by ACT
   2. Haemostasis Management System plus (HMS plus, Medtronic®)
   3. ACT Plus (Medtronic®)
   4. Hemochron® Signature Elite
   5. Hemochron® Response
   6. i-STAT (Abbot®)
   7. Other (explain) …

**Page 7 Comments**

1. Do you have any additional comments regarding your antithrombotic strategies during NCAP? …

Abbreviations: CEA = carotid endarterectomy, EVAR/TEVAR = endovascular aneurysm repair and/or thoracic endovascular aneurysm repair, FEVAR/BEVAR = fenestrated endovascular aneurysm repair and/or branched endovascular aneurysm repair, IV = intravenous, LMWH = low molecular weight heparin, ACT = activated clotting time, APTT = activated partial thromboplastin time, NCAP = non-cardiac arterial procedures.

**Supplementary table S2.** Overview of the included vascular clinical specialists per country.

| **Country** | **No. of participants** | **Country** | **No. of participants** |
| --- | --- | --- | --- |
| Albania | 2 | Italy | 40 |
| Algeria | 2 | Lithuania | 1 |
| Argentina | 1 | Luxembourg | 4 |
| Australia | 6 | Netherlands | 52 |
| Austria | 22 | Norway | 11 |
| Bahrain | 1 | Oman | 1 |
| Belgium | 9 | Poland | 5 |
| Brazil | 3 | Portugal | 20 |
| Canada | 2 | Reunion | 1 |
| Colombia | 1 | Romania | 4 |
| Croatia | 4 | Russia | 3 |
| Cyprus | 1 | Saudi Arabia | 1 |
| Czech Republic | 1 | Serbia | 19 |
| Denmark | 14 | Spain | 4 |
| Egypt | 1 | Sweden | 7 |
| El Salvador | 1 | Switzerland | 16 |
| Finland | 12 | Thailand | 1 |
| France | 69 | Turkey | 5 |
| Germany | 15 | Ukraine | 1 |
| Greece | 23 | United Kingdom | 21 |
| Hungary | 6 | United States | 8 |
| India | 1 | Yemen | 2 |
| Ireland | 12 |  |  |
| **Total** | **436** | | |

**Supplementary table S3**. Activated clotting time measurements after heparin administration per type of non-cardiac arterial procedure.

|  | **First ACT after heparin**  **(min)** | | | | | | **Follow up ACT after reaching target value (min)** | | | | | |
| --- | --- | --- | --- | --- | --- | --- | --- | --- | --- | --- | --- | --- |
| **Procedure** | **No. (% of ACT users)*** | **1 – 5** | **6 – 15** | **16 – 30** | **31 – 60** | **> 60** | **No. (% of ACT users)*** | **< 30** | **30** | **31 – 59** | **60** | **> 60** |
| CEA | 81 (72) | 35 | 20 | 18 | 6 | 2 | 80 (71) | 3 | 35 | 6 | 30 | 6 |
| EVAR/TEVAR | 93 (87) | 34 | 24 | 26 | 6 | 3 | 92 (86) | 4 | 38 | 5 | 34 | 11 |
| FEVAR/BEVAR | 91 (88) | 19 | 30 | 27 | 13 | 2 | 90 (87) | 2 | 36 | 4 | 39 | 9 |
| OAR | 85 (87) | 33 | 19 | 21 | 9 | 3 | 84 (86) | 3 | 38 | 3 | 33 | 7 |
| PAD surgery | 80 (84) | 32 | 17 | 20 | 8 | 3 | 79 (83) | 4 | 36 | 5 | 28 | 6 |

Abbreviations: ACT = activated clotting time, CEA = carotid endarterectomy, EVAR/TEVAR = endovascular aneurysm repair and/or thoracic endovascular aneurysm repair, FEVAR/BEVAR = fenestrated endovascular aneurysm repair and/or branched endovascular aneurysm repair, OAR = open abdominal aortic repair, PAD = peripheral arterial disease.

*Measurement strategies are depicted as percentage of total of ACT users per procedure.
