# Supplementary material for: Strategies for Antithrombotic Management During Non-cardiac Arterial Procedures: Results of the International ACTION Survey
Source: EJVES Vasc Forum. 2025 Apr 2;64:8–15. doi: 10.1016/j.ejvsvf.2025.01.005 (PMC12269446; doi:10.1016/j.ejvsvf.2025.01.005)
Supplement: Multimedia component 2 [file mmc2.docx]

**Collaborators**

**European Vascular Research Collaborative steering committee:** Charlotte Arendt, Gert

J. de Borst, Albert Busch, Caroline Caradu, Alexander Croo, Vaiva Dabravolskaité, Mariam

Darwish, Mario D’Oria, Harm P. Ebben, Florian Enzmann, Qasam M. Ghulam, Alexander

Gombert, Alexandra Gratl, Bergros K. Johannesdottir, Angelos Karelis, Aoife Kiernan,

Leszek Kukulski, Fabien Lareyre, Ryan Gouveia e Melo, Cecilie Markvard Møller,

Panagiotis Doukas, Nikolaos Patelis, Konstantinos Spanos, Paolo Spath, Martin Teraa, Bich

L. Tran, Christian Zielasek, Petar Zlatanovic, Kak Khee Yeung.

Contributions: survey construction, survey deployment, review & editing of final manuscript.

**ACTION-1 Research Collaborative:** Liliane C. Roosendaal, Jan D. Blankensteijn, Hessel C.J.L. Buscher, Daniël Eefting, Bram Fioole, Jan M.M. Heyligers, Rutger J. Hissink, Rigo Hoencamp, Mark J.W. Koelemay, Rogier H.J. Kropman, Lijckle van der Laan, Susan Lemson, Maurice E.N. Pierie, Boudewijn L. Reichmann, Michel M.P.J. Reijnen, Jan van Schaik, Peter M. Schlejen, Joep A.W. Teijink, Edith M. Willigendael, Clark J. Zeebregts, Arno M. Wiersema.

Contributions: survey construction, survey deployment, review & editing of final manuscript.
